# Tocilizumab promotes repair of spinal cord injury by facilitating the restoration of tight junctions between vascular endothelial cells

**DOI:** 10.1186/s12987-022-00399-9

**Published:** 2023-01-09

**Authors:** Yang Luo, Fei Yao, Yi Shi, Zhenyu Zhu, Zhaoming Xiao, Xingyu You, Yanchang Liu, Shuisheng Yu, Dasheng Tian, Li Cheng, Meige Zheng, Juehua Jing

**Affiliations:** 1 Department of Orthopaedics & Spine Surgery, The Second Hospital of Anhui Medical University, Anhui Medical University, Hefei, 230601 China; 2 Institute of Orthopaedics, Research Center for Translational Medicine, The Second Hospital of Anhui Medical University, Anhui Medical University, Hefei, 230601 China; 3 Department of Orthopedic Disease and Oncology Surgery, The First Affiliated Hospital of Anhui Medical University, Anhui Medical University, Hefei, 230601 China

**Keywords:** Spinal cord injury, Tocilizumab, Blood-spinal cord barrier, Tight junctions, Axon regeneration

## Abstract

**Background:**

Our previous study demonstrated that M1 macrophages could impair tight junctions (TJs) between vascular endothelial cells by secreting interleukin-6 (IL-6) after spinal cord injury (SCI). Tocilizumab, as a humanized IL-6 receptor (IL-6R) monoclonal antibody approved for the clinic, has been applied in the treatment of neurological diseases in recent years, but the treatment effect of Tocilizumab on the TJs restoration of the blood-spinal cord barrier (BSCB) after SCI remains unclear. This study aimed to explore the effect of Tocilizumab on the restoration of TJs between vascular endothelial cells and axon regeneration after SCI.

**Methods:**

In this study, the mouse complete spinal cord crush injury model was used, and Tocilizumab was continuously injected intrathecally until the day of sample collection. A PBS injection in the same location was included as a control. At 14 days postinjury (dpi) and 28 dpi, spinal cord tissue sections were examined via tissue immunofluorescence. The Basso Mouse Scale (BMS) scores and footprint analysis were used to verify the effect of Tocilizumab on the recovery of motor function in mice after SCI.

**Results:**

We demonstrated that depletion of macrophages has no effect on axon regeneration and motor functional recovery after SCI, but mice subjected to Tocilizumab showed a significant increase in axon regeneration and a better recovery in motor function during the chronic phase after SCI. Moreover, our study demonstrated that at 14 and 28 dpi, the expression of claudin-5 (CLDN5) and zonula occludens-1 (ZO-1) between vascular endothelial cells was significantly increased and the leakage of BSCB was significantly reduced in the injured core after daily intrathecal injection of Tocilizumab. Notably, the infiltration of CD68^+^ macrophages/microglia and the formation of fibrotic scar were decreased in the injured core after Tocilizumab treatment. Tocilizumab treatment could effectively reduce the IL-6 expression in macrophages in the injured core.

**Conclusion:**

The application of Tocilizumab to antagonize IL-6R can effectively reduce the expression of IL-6 in macrophages and facilitate TJs restoration of the BSCB, which is beneficial for axon regeneration and motor functional recovery after SCI. Hence, Tocilizumab treatment is a potential therapeutic strategy for SCI.

**Supplementary Information:**

The online version contains supplementary material available at 10.1186/s12987-022-00399-9.

## Introduction

The blood-spinal cord barrier (BSCB) is damaged during the primary injury stage of spinal cord injury (SCI) [1]. In the secondary injury stage, the repair of the BSCB is hindered due to continuous inflammation, which conversely leads to the persistent leakage of the BSCB and aggravates secondary injury [1, 2]. BSCB helps to regulate the molecular exchange between the peripheral circulatory system and the spinal cord parenchyma, thereby contributing to spinal homeostasis. Therefore, the repair of the BSCB can effectively prevent the development of secondary injury and play an important role in reducing tissue damage after SCI. Multiple cell types and structures are responsible for the barrier function of the BSCB, including the basement membrane, pericytes, astrocyte endfeet, nonfenestrated capillary endothelial cells and intercellular connections [3]. Endothelial cells are connected to each other by tight junctions (TJs) and adhesive junctions [4]. TJs are mainly composed of transmembrane proteins such as claudins, occludin, and intracellular proteins such as Zonula Occludens-1 (ZO-1) [5]. A major function of TJs is to form a permeability barrier in tight endothelia and to determine the selective permeability in leaky barriers where the paracellular diffusion pathway is largely restricted [6]. Damage to tight junctions is one of the important causes of BSCB leakage after SCI. Our previous study showed that macrophages that aggregated in the lesion epicenter after SCI could impair TJs between vascular endothelial cells by secreting interleukin-6 (IL-6), resulting in persistent leakage of the BSCB [7]. Intraperitoneal injection of MR16-1 to antagonize the IL-6 receptor (IL-6R) can promote motor functional recovery in mice after SCI. The mechanism may be related to reducing the activation of astrocytes or promoting an alternative pathway of macrophage activation [8]. Antagonizing IL-6R after SCI reduces the excessive inflammatory response at the injured site [9]. In addition, blocking IL-6R with an IL-6R antibody can ease pain after SCI in rats [10]. These studies suggest that inhibition of IL-6/IL-6R pathway may be beneficial for promoting tissue repair and functional recovery after SCI.

Tocilizumab, a humanized IL-6R monoclonal antibody, was approved by the U.S. Food and Drug Administration (FDA) in 2010 for the clinical treatment of rheumatoid arthritis patients [11]. Although Tocilizumab is a humanized monoclonal antibody against IL-6R, its effects in mice have been widely reported [12–14]. In recent years, several studies have reported the application of Tocilizumab in neurological diseases [15, 16]. However, experimental treatment with Tocilizumab for BSCB restoration after SCI has not been reported. Our study aims to demonstrate the effect of blocking IL-6R by intrathecal injection of Tocilizumab on the restoration of TJs between vascular endothelial cells and investigate the therapeutic effect of Tocilizumab in SCI model.

In this study, we demonstrated that intrathecal injection of Tocilizumab facilitated the restoration of TJs between vascular endothelial cells and reduced the leakage of the BSCB at 14 days postinjury (dpi) and 28 dpi after SCI. Moreover, the aggregation of macrophages/microglia and the formation of fibrotic scar in the injured core in SCI could be reduced after Tocilizumab treatment. The IL-6 expression in macrophages in the injured core was significantly reduced after Tocilizumab treatment. We further verified that the application of Tocilizumab could effectively promote axon regeneration and motor functional recovery in the chronic phase of SCI.

## Methods

### Animals

All animal experiments were approved by the Animal Ethics Committee of Anhui Medical University (Approval No. LLSC20160052). Female C57BL/6J mice weighing 18–22 g were purchased from the Animal Experiment Center of Anhui Medical University. A total number of 78 mice were used in this study. All mice were bred with the appropriate temperature and humidity under a 12 h light and darkness cycle, and food and water were readily available.

### Mouse complete spinal cord crush injury model and tocilizumab treatment

The mice were anaesthetized by intraperitoneal injection of 50 mg/kg pentobarbital sodium. The T10 segment of the spinal cord was exposed after laminectomy. The severe crush SCI model was established by clamping the spinal cord at the T10 level using calibrated Dumont No. 5 forceps (11252-20, Fine Science Tools, Germany) for 5 s from two sides. The appearance of a clear red clamp trace at the spinal cord clamping site and the vigorous swinging of the lower limbs and tail of the mouse indicated that the model was successfully established. The lamina was removed from mice in the sham group without clamping the spinal cord. The incisions were closed carefully layer by layer. Anti-infection and auxiliary urination nursing were performed twice a day after SCI.

Tocilizumab was diluted with phosphate buffered saline (PBS) to a concentration of 1 µg/µl. Immediately after injury, 10 µl diluted Tocilizumab was intrathecally injected via a microsyringe (1701, Hamilton, United States). The insertion site was in the dorsal midline of the lumbar 5–6 intervertebral space as previously reported [17]. Successful insertion into the intrathecal space was confirmed by observing an evident tail flick. The PBS group was injected with the same volume of human IgG in PBS. Injections were performed once a day at the same time point until the day of sample collection.

### Immunofluorescence staining

After anaesthetization with pentobarbital sodium, thoracotomy was performed. After cardiac perfusion with cold PBS followed by 4% paraformaldehyde (PFA, Servicebio, China), the injured spinal cord tissue containing the injured core (5 mm) was removed and fixed in 4% PFA for 5 h and in 30% sucrose for 24 h before embedding in tissue freezing media (Sakura, United States). Sixteen-micrometer-thick sections were prepared on a cryostat (NX50, Thermo Fisher, United States). All sections were obtained in sagittal view. The sections were washed with PBS 3 times and blocked in PBS with 5% donkey serum albumin (DSA, SL050, Solarbio, China) and 0.3% Triton X-100 (T8200, Solarbio, China) for 1 h at room temperature. The tissue sections were incubated at 4 °C overnight with primary antibodies, including goat anti-CD31 (1:200, AF3628, R&D Systems, United States), mouse anti-claudin-5 (CLDN5, 1:100, 35-2500, Invitrogen, United States), rabbit anti-ZO-1 (1:100, 61-7300, Invitrogen, United States), goat anti-platelet derived growth factor receptor β (PDGFRβ, 1:100, AF1042, R&D Systems, United States), rabbit anti-neurofilament (NF, 1:500, Ab207176, Abcam, United States), goat anti-5-hydroxytryptamine (5-HT, 1:100, 20079, Immunostar, United States), rabbit anti-glial fibrillary acidic protein (GFAP, 1:100, 16825-1-AP, Proteintech, China), rat anti-GFAP (1:200, 13-0300, Invitrogen, United States), rabbit anti-growth-associated protein 43 (GAP-43, 1:100, 16971-1-AP, Proteintech, China), rabbit anti-NeuN (1:500, ab177487, Abcam, United States), rat anti-CD68 (1:500, MCA1957, AbD Serotec, United Kingdom), rabbit anti-fibrinogen (1:100, 15841-1-AP, Proteintech, China), rabbit anti-IL-6 (1:100, 12912, Cell Signaling Technology, United States), and rat anti-F4/80 (1:100, 14-4801-82, Invitrogen, United States). The sections were incubated at 37 °C in the dark for 1 h with the following secondary antibodies: donkey anti-rabbit Alexa Fluor 488, donkey anti-mouse Alexa Fluor 488, donkey anti-goat Alexa Fluor 594, and donkey anti-rat Alexa Fluor 488 (1:500, A-21206, A-21202, A-21209, A-11058, Invitrogen, United States). Finally, the sections were sealed with an anti-fluorescence quencher (P0126, Beyotime Biotechnology, China). Immunofluorescence images were obtained using a fluorescence microscope system (Axio Scope A1, Zeiss, Germany) with the same light intensity and filtering.

### Hematoxylin and eosin (HE) staining

HE staining was performed with a HE staining kit (C0105 M, Beyotime, China). Tissue sections were obtained as described above. The sections were fixed in an oven at 50 ℃ for 30 min followed by washing with PBS 3 times. Then the sections were stained with hematoxylin solution for 4 min and washed in distilled water for 10 min. Differential solution was added for 10 s, and sections were washed with water for 10 min. Sections were dehydrated in 70%, 80%, and 90% alcohol successively. Then, the sections were stained with eosin staining solution for 2–3 min. The dye was removed, and the sections were washed with absolute ethyl alcohol and xylene for 15 min. Finally, the sections were sealed with neutral gum and observed under a microscope system (Axio Scope A1, Zeiss, Germany).

### Depletion of macrophages in the SCI lesion site

To deplete macrophages, undiluted PBS liposomes (PL) or clodronate liposomes (CL) (2 µl, #CLD-8909, Encapsula NanoSciences, United States) were stereotaxically injected into the lesion site at 5 dpi and the effect of macrophage depletion was assessed at 14 dpi [18]. The density of macrophage coverage in the injuried core of each section was determined by quantifying the percent of the contoured area covered by CD68 staining using the Measure and Analyse Particles functions in ImageJ software (NIH, United States).

### Quantification

Immunostaining of CD31 was used to identify endothelial cells. ZO-1 and CLDN5 were used to identify TJs. For analysis of the vessels with TJ proteins, 4–6 sagittal sections per animal were immunostained and images were taken at the lesion epicenter and penumbra using 10× and 40× objectives. The ratio of the number ZO-1 or CLDN5 colabelled with CD31^+^ vessels to the total number of CD31^+^ vessels represented the percent of the vessels with TJ proteins under a 10× objective.

Immunostaining of NF was used to identify axons and GFAP was used to identify astrocytes. For quantification of the NF^+^ axons, 4–6 sagittal sections per animal were immunostained and images were taken at the lesion epicenter using a 10× objective. Then the number of NF^+^ axons greater than 1 mm in length within the GFAP^−^ area was counted and normalized to the GFAP^−^ area. Similarly, the number of GAP-43^+^ axons within the GFAP^−^ area was counted and normalized to the GFAP^−^ area. For quantification of the 5-HT^+^ axons, images were taken 0.5 mm distal to the lesion epicenter using a 20× objective and the number of 5-HT^+^ axons was counted. For quantification of NeuN^+^ neurons, images were taken 1.5 mm distal to the lesion epicenter using a 4× objective, and the number of NeuN^+^ neurons was counted.

To evaluate the area of fibrotic scar, 4–6 sagittal sections per animal were immunostained and the immunoreactivities of PDGFRβ were normalized to the area of the spinal cord segment spanning the injured core in a 4× objective. Similarly, the CD68^+^ area was normalized to the area of the spinal cord segment spanning the injured core in a 4× objective. Immunostaining of IL-6 and F4/80 was used to identify the effect of Tocilizumab treatment on IL-6 expression in macrophages. The ratio of the number of IL-6 colabelled with F4/80^+^ macrophages to the total number of F4/80^+^ macrophages were represented as the proportion of IL-6^+^ macrophages from 4 to 6 sagittal sections per animal under a 10× objective.

### Behavioral analysis

The observer of the behavioral analysis was blinded to the treatment. Motor function was evaluated with the Basso Mouse Scale (BMS) score. Two experienced examiners evaluated each animal for 4 min and assigned a score for each hind limb. The BMS score of each mouse was assessed at 0, 3, 7, 14, 21, and 28 dpi. For the footprint analysis, the forelimb and hindlimb of the mice were dipped in red and green dyes at 28 dpi. A narrow runway (80 cm long, 4 cm wide) was lined with white paper as the mice walked across. The stride length was defined as the distance from the start to the end of a step with the back paw. Stride width was defined as the distance from the left outermost toe to the right outermost toe. Paw rotation was defined as the angle between the axis of the back paw and the midline axis of the body. All measurements were taken on each side for three consecutive steps and averaged.

### Statistical analysis

All measurements, analyses, and statistics were performed blindly. The data were presented as the mean ± standard error of the mean (SEM). Statistical significance among multiple groups was determined by one-way or two-way analysis of variance followed by Tukey’s post hoc test using Prism 7.0 software (GraphPad, United States). *P* < 0.05 was considered as statistically significant.

## Results

### Tocilizumab treatment ameliorates motor functional recovery after SCI

**Fig. 1:**
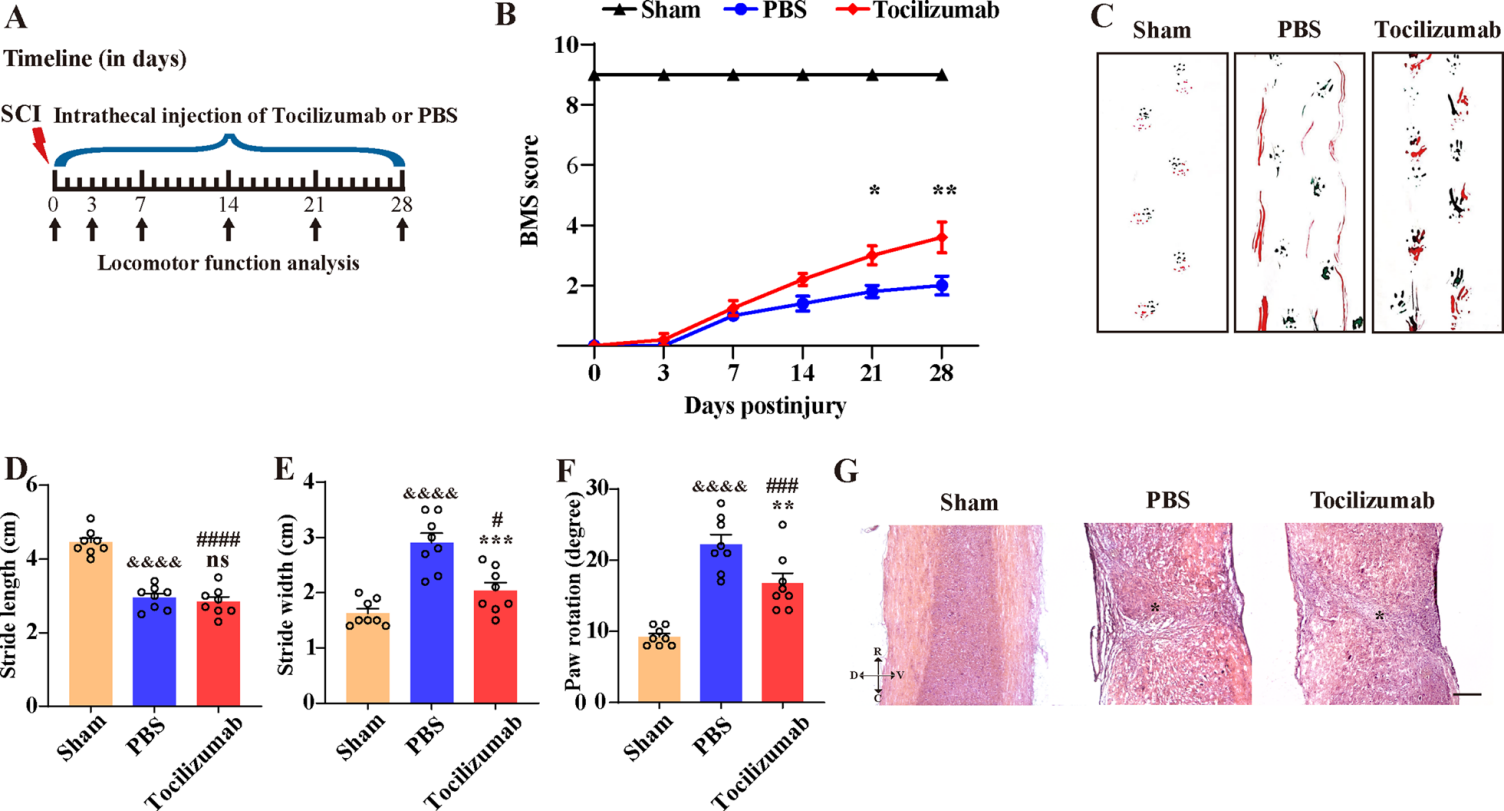
Tocilizumab ameliorates motor function recovery after SCI. **A** A schematic illustration of the Tocilizumab injection protocol after SCI. After the operation, Tocilizumab or PBS was intrathecally injected daily at the same time point until the day of sample collection. **B** The time course of functional recovery assessed by the BMS score in the sham, PBS, and Tocilizumab groups. **C**–**F** The results of the footprint analyses in the sham, PBS, and Tocilizumab groups at 28 dpi after SCI. Data are presented as the mean ± SEM (n = 8 in each group). **p < 0.01 and ***p < 0.001 (Tocilizumab vs. PBS). ^#^*p* < 0.05, ^###^*p* < 0.001, ^####^*p* < 0.0001 (Tocilizumab vs. sham). ^&&&&^*p* < 0.0001 (PBS vs. sham). ns, no significance (Tocilizumab vs. PBS). **G** Representative images of HE staining in the sham group and at 28 dpi among the PBS and Tocilizumab groups. Asterisks indicate the injured epicenter. Scale bar: 200 μm. D, V, R, and C represent the dorsal, ventral, rostral, and caudal sides of the injury, respectively

Our previous study demonstrated that the depletion of macrophages facilitated the restoration of TJs between vascular endothelial cells and reduced BSCB leakage in the lesion epicenter after SCI [7]. To further confirm macrophage depletion on axon regeneration and functional recovery, intraspinal injection of CL was performed at 5 dpi and the clearance effect was detected by immunofluorescence staining of CD68 at 14 dpi (Additional file 1: Fig. S1A, B). Immunofluorescence staining of NF and BMS scores were carried out to assess axon regeneration and functional recovery. We found that macrophage depletion had no evident effect on axon regeneration and motor functional recovery after SCI (Additional file 1: Fig. S1C–I). Considering the complex functions of macrophages with different phenotypes and our previous study that showed that M1 macrophages impaired TJs between vascular endothelial cells by secreting IL-6 [7], we further blocked IL-6R specifically by Tocilizumab to verify its effect on motor functional recovery after SCI (Fig. 1A). The results showed that there was no significant difference in the BMS score of the Tocilizumab treatment group compared with the PBS group at 3, 7, and 14 dpi, while the BMS score of mice after continuous Tocilizumab treatment were significantly higher than those in the PBS group at 21 and 28 dpi (Fig. 1B). The footprint analysis revealed significantly better motor functional recovery of mice at 28 dpi after Tocilizumab treatment compared with PBS group (Fig. 1C–F). HE staining was performed to evaluate the structure of the injured spinal cord among the sham, PBS, and Tocilizumab groups (Fig. 1G). The HE staining results showed a clear distinction between white matter and gray matter in the sham group. There was a clear boundary between the injury site and the uninjured spinal cord tissue, and the injury area of the Tocilizumab group was significantly smaller than that of the PBS group. These results suggested that the administration of Tocilizumab could promote functional recovery and tissue repair in the chronic phase of SCI in mice.

### Tocilizumab treatment promotes the restoration of TJs between vascular endothelial cells after SCI

**Fig. 2:**
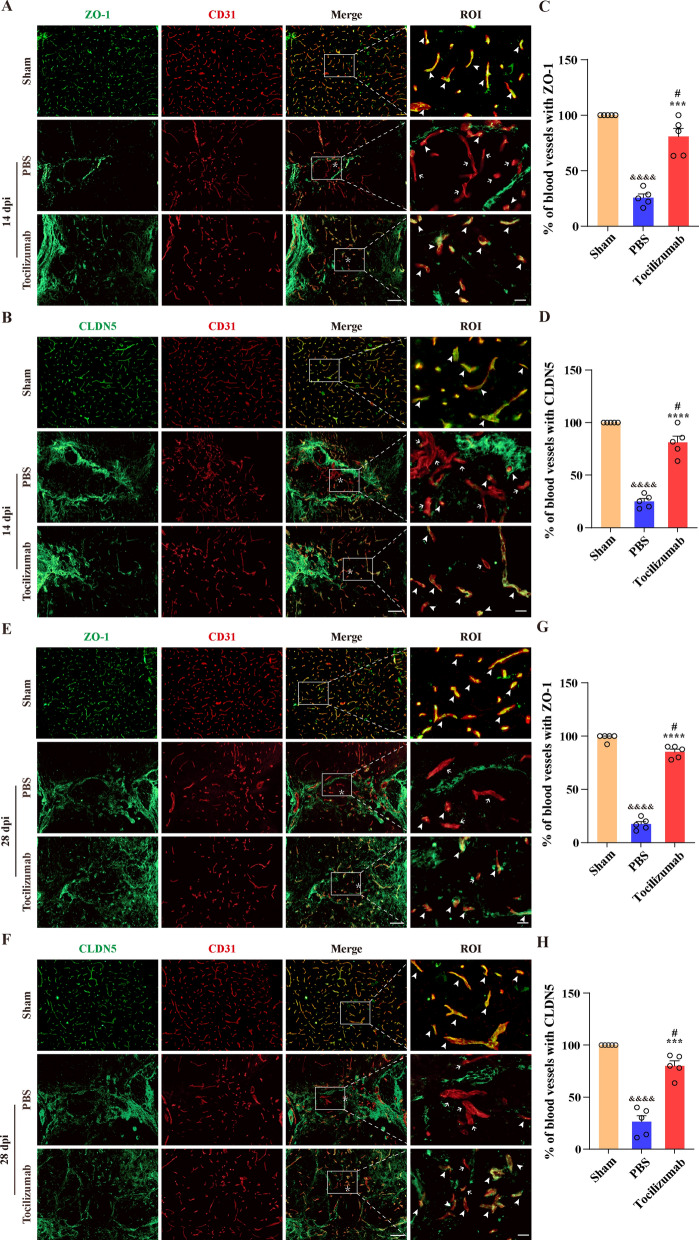
Tocilizumab facilitates the restoration of TJs between vascular endothelial cells after SCI. **A**, **B**, **E** and **F** Representative images of sham, PBS, and Tocilizumab group mice spinal cord are stained for ZO-1 or CLDN5 (green) and CD31 (red) at 14 (**A**, **B**) and 28 dpi (**E**, **F**). The region of interest (ROI) shows a higher magnification of the left boxed region, which exhibits little colocalization of CD31 and ZO-1 (**A**,  **E**) or CLDN5 (**B**, **F**) in the injured core in the PBS group, while most CD31 colocalizes with ZO-1 (**A**, **E**) or CLDN5 (**B**, **F**) in the Tocilizumab group and the sham group. Arrowheads indicate ZO-1^+^ (**A**, **E**) or CLDN5^+^ (**B**, **F**) blood vessels, arrows indicate ZO-1^−^ (**A**, **E**) or CLDN5^−^ (**B**, **F**) blood vessels. Asterisks indicate the injured core. Scale bars: low magnification, 100 μm; higher magnification, 20 μm. (**C**, **D**, **G** and **H**) Quantitative analysis of the percentage of endothelial ZO-1 (**C**, **G**) or CLDN5 (**D**, **H**) in sham group and in the injured core in the PBS and Tocilizumab groups at 14 (**C**, **D**) and 28 dpi (**G**, **H**). All images show sagittal sections. Data are presented as the mean ± SEM (n = 5 in each group). ***p < 0.001 and ****p < 0.0001 (Tocilizumab vs. PBS). ^#^*p* < 0.05 (Tocilizumab vs. sham). ^&&&&^*p* < 0.0001 (PBS vs. sham)

We have demonstrated that the application of an IL-6 neutralizing antibody can inhibit the destruction of TJs between vascular endothelial cells in vitro [7]. To further evaluate the effect of IL-6 on TJs restoration between vascular endothelial cells after SCI, we injected Tocilizumab intrathecally to block IL-6R as described above. Immunofluorescence staining results showed that all CD31^+^ blood vessels expressed TJs (ZO-1 and CLDN5, Fig. 2A–H) in sham group. While the colocalization of CD31^+^ blood vessels and ZO-1^+^ TJ or CLDN5^+^ TJ in the injured core was increased significantly at 14 and 28 dpi after Tocilizumab treatment compared with the PBS group, although the proportion of vascular endothelial cells co-located with TJs was still significantly lower than that in the sham group (Fig. 2A–H). These findings revealed that Tocilizumab treatment can promote the restoration of TJs between vascular endothelial cells after SCI.

### Tocilizumab treatment reduces BSCB leakage in the lesion epicenter after SCI

**Fig. 3:**
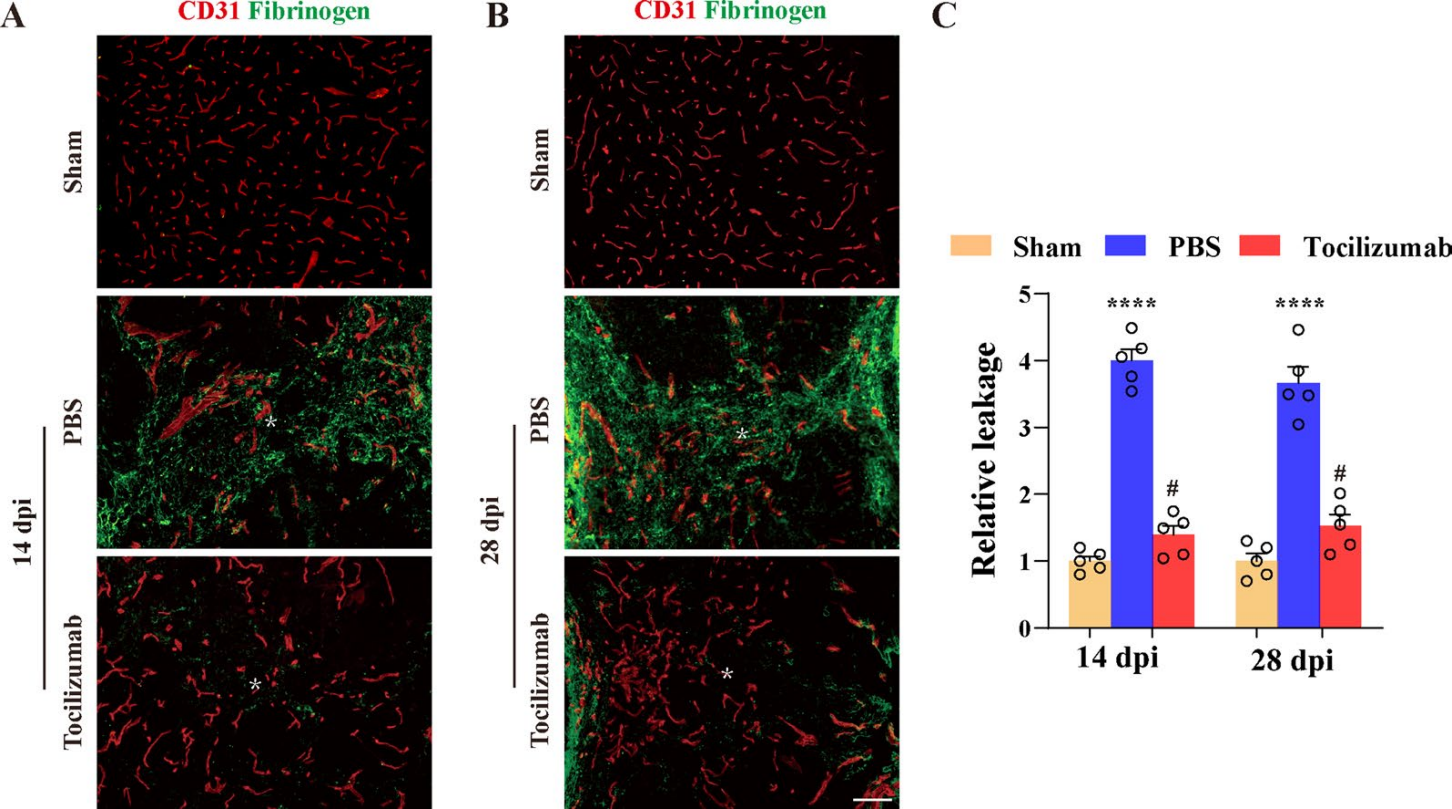
Tocilizumab decreases BSCB leakage in the injured epicenter after SCI. **A**, **B** Double immunostaining of fibrinogen (green) and CD31 (red) was used to examine BSCB permeability in the injured core at 14 (**A**) and 28 dpi (**B**) and in normal spinal cord tissue (sham). Fibrinogen leakage was severe in the PBS group in the injured core, while no obvious fibrinogen leakage is observed in the Tocilizumab and sham groups. Asterisks indicate the injured epicenter. Scale bar: 100 μm. **C** Quantitative analysis of the relative fluorescence intensity of leaky fibrinogen as shown in **A** and **B**. All images show sagittal sections. Data are presented as the mean ± SEM (n = 5 in each group). ****p < 0.001 (PBS vs. sham and Tocilizumab). ^#^*p* < 0.05 (Tocilizumab vs. sham)

We detected fibrinogen leakage in spinal cord tissue by immunofluorescence staining and quantified the fluorescence intensity to assess BSCB permeability after Tocilizumab treatment. Our results showed that there was obvious fibrinogen leakage in the lesion epicenter at 14 and 28 dpi in the PBS group (Fig. 3A, B), indicating that a large amount of fibrinogen leaked from blood vessels to the spinal cord parenchyma after SCI, while there was only a small amount of fibrinogen leakage in the lesion epicenter at 14 and 28 dpi in the Tocilizumab treatment group (Fig. 3A–C). In contrast, the sham group spinal cord had no significant fibrinogen leakage (Fig. 3A–C). These results demonstrated that Tocilizumab treatment reduced BSCB leakage in the lesion epicenter during the chronic phase of SCI.

### Tocilizumab treatment inhibits inflammation and fibrotic scarring after SCI

**Fig. 4:**
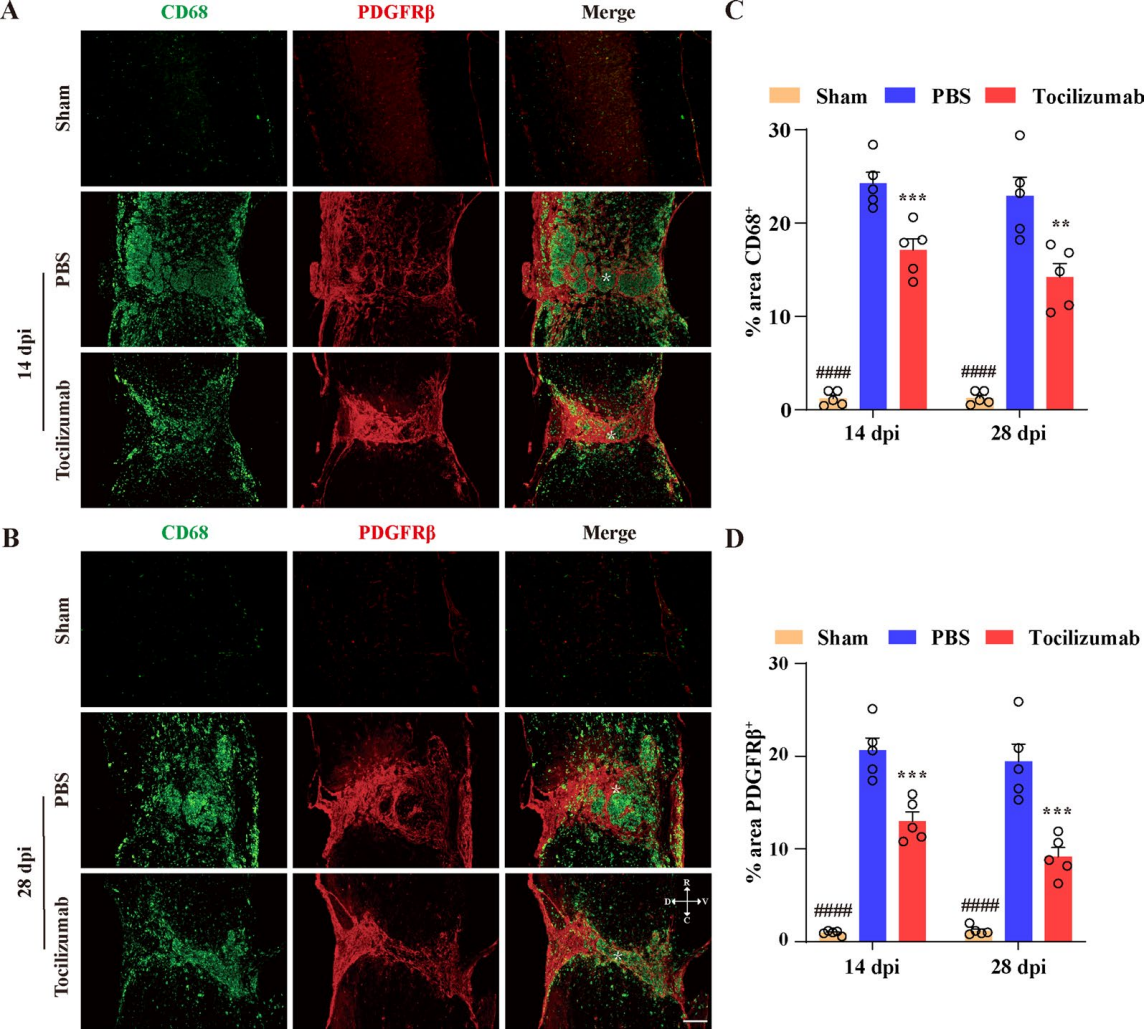
Tocilizumab reduces macrophage/microglia inflammation and the formation of fibrotic scar after SCI. **A**, **B** Representative images of the spinal cords of the sham, PBS, and Tocilizumab groups are stained for CD68 (green) and PDGFRβ (red) at 14 (**A**) and 28 dpi (**B**), which exhibit fewer CD68^+^ macrophages/microglia and PDGFRβ^+^ fibroblasts in the injured core in the Tocilizumab group than in the PBS group. Asterisks indicate the injured core. Scale bars: 200 μm. D, V, R, and C represent the dorsal, ventral, rostral, and caudal sides of the injury, respectively. **C**, **D** Quantitative analysis of the CD68^+^ area (**C)** and PDGFRβ^+^ area (**D**) in the lesion epicenter at 14 and 28 dpi. Data are presented as the mean ± SEM (n = 5 in each group). **p < 0.01 and ***p < 0.001 (Tocilizumab vs. PBS). ^####^*p* < 0.0001 (sham vs. PBS and Tocilizumab)

Disruption of the BSCB causes the infiltration of immune cells in the spinal cord parenchyma, which can be reduced after BSCB repair [19, 20]. Immune cells and proteins infiltrating the spinal cord parenchyma are reduced after BSCB repair, which promotes the formation of fibrotic scar [21, 22]. IL-6 has been known to promote macrophage infiltration in the inflammatory response [23]. To further confirm the effect of Tocilizumab treatment on the infiltration of macrophages and fibrotic scarring, we performed immunofluorescence staining of CD68 and PDGFRβ in injured spinal cord tissues at 14 and 28 dpi. As shown in Fig. 4A–C, there were few CD68^+^ macrophages/microglia and PDGFRβ^+^ pericytes in the sham group spinal cords. Notably, the CD68^+^ macrophages/microglia and PDGFRβ^+^ fibrotic scar in the lesion epicenter significantly increased at 14 and 28 dpi, as shown in the PBS group. Tocilizumab treatment could significantly reduce the area CD68^+^ macrophages/microglia compared with the PBS group, indicating that macrophage/microglia infiltration in the lesion epicenter could be reduced by Tocilizumab after SCI. Similarly, we found that the area of PDGFRβ^+^ fibrotic scar in the Tocilizumab treatment group was significantly smaller than that in the PBS group at 14 and 28 dpi (Fig. 4A, B and D), which suggested that Tocilizumab could decrease the formation of fibrotic scar after SCI. Hence, these results indicated that Tocilizumab treatment inhibited inflammation and fibrotic scarring after SCI.

### Tocilizumab treatment facilitates axon regeneration after SCI

**Fig. 5:**
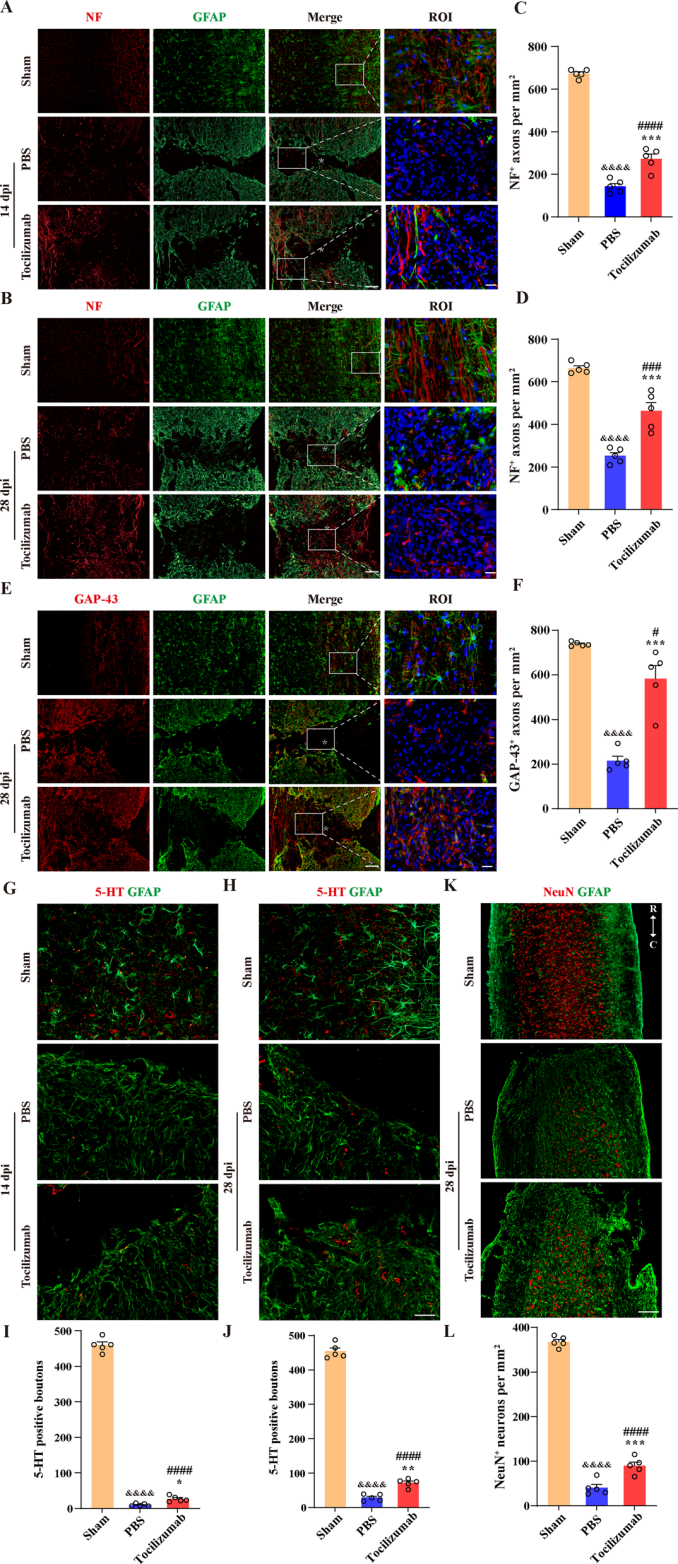
Tocilizumab facilitates axon regeneration after SCI. **A**, **B**, **G** and **H** Representative images of sham, PBS, and Tocilizumab groups mice spinal cord are stained for NF (red, **A** and **B**), 5-HT (red, **G** and **H**), and GFAP (green, **A**, **B**, **G** and **H** ) at 14 (**A**, **G**) and 28 dpi (**B**, **H**), which exhibit fewer NF^+^ axons in the injured core and 5-HT^+^ axons 0.5 mm distal to the injured core in the PBS group compared to sham group, while there are more NF^+^ axons in the injured core and 5-HT^+^ axons 0.5 mm distal to the injured core in the Tocilizumab group than in the PBS group. **C**, **D**, **I** and **J** Quantitative analysis of the number of NF^+^ axons (**C**, **D**) and 5-HT^+^ axons (**I**, **J**) at 14 (**C**, **I**) and 28 dpi (**D**, **J**). **E**, **K** Representative images of the spinal cords of the sham, PBS, and Tocilizumab groups are stained for GAP-43 (red, **E**), NeuN (red, **K**), and GFAP (green, **E** and **K**) at 28 dpi, which exhibit fewer GAP-43^+^ axons in the injured core and NeuN ^+^ neurons 1.5 mm distal to the injured core in the PBS group compared to sham group, while there are more GAP-43^+^ axons in the injured core and NeuN ^+^ neurons 1.5 mm distal to the injured core in the Tocilizumab group compared to PBS group. **F**, **L** Quantitative analysis of the number of GAP-43^+^ axons (**F**) and NeuN^+^ neurons (**L**) in the GFAP^−^ region at 28 dpi. The ROI shows a higher magnification of the left boxed region (**A**, **B** and **E**). Asterisks indicate the injured core. Scale bars: 100 μm (low magnification, **A**, **B** and **E**), 20 μm (higher magnification, **A**, **B** and **E**), 50 μm (**G**, **H**), and 200 μm (K). R, and C represent the rostral and caudal sides of the injury, respectively. Data are presented as the mean ± SEM (n = 5 in each group). *p < 0.05, **p < 0.01, and ***p < 0.001 (Tocilizumab vs. PBS). ^#^*p* < 0.05, ^###^*p* < 0.001 and ^####^*p* < 0.0001 (Tocilizumab vs. sham). ^&&&&^*p* < 0.0001 (PBS vs. sham)

BSCB leakage, the inflammatory response, and fibrotic scarring are major obstacles to axon regeneration after SCI [24, 25]. Tocilizumab treatment can promote BSCB repair and reduce the inflammatory response and fibrotic scarring after SCI. Therefore, we next assessed the effect of Tocilizumab on axon regeneration after SCI. Immunofluorescence staining results showed that in the lesion epicenter the density of NF^+^ axons at 14 and 28 dpi and GAP-43^+^ axons at 28 dpi increased significantly after Tocilizumab treatment (Fig. 5A–F). In addition, the number of 5-HT^+^ axons on the caudal side of the lesion epicenter at 14 and 28 dpi and NeuN^+^ neurons on the caudal side of the lesion epicenter at 28 dpi in the Tocilizumab treatment group was greater than that in the PBS group (Fig. 5E–H). These findings suggested that continuous administration of Tocilizumab can effectively promote axon regeneration in the chronic phase of SCI. However, the density and the number of axons were still significantly lower in Tocilizumab treatment group than in sham group (Fig. 5A–L), revealing that the restoration of axons could not reach to normal level by Tocilizumab treatment at 28 dpi.

### Tocilizumab treatment decreases the expression of IL-6 in macrophages in the lesion epicenter after SCI

**Fig. 6:**
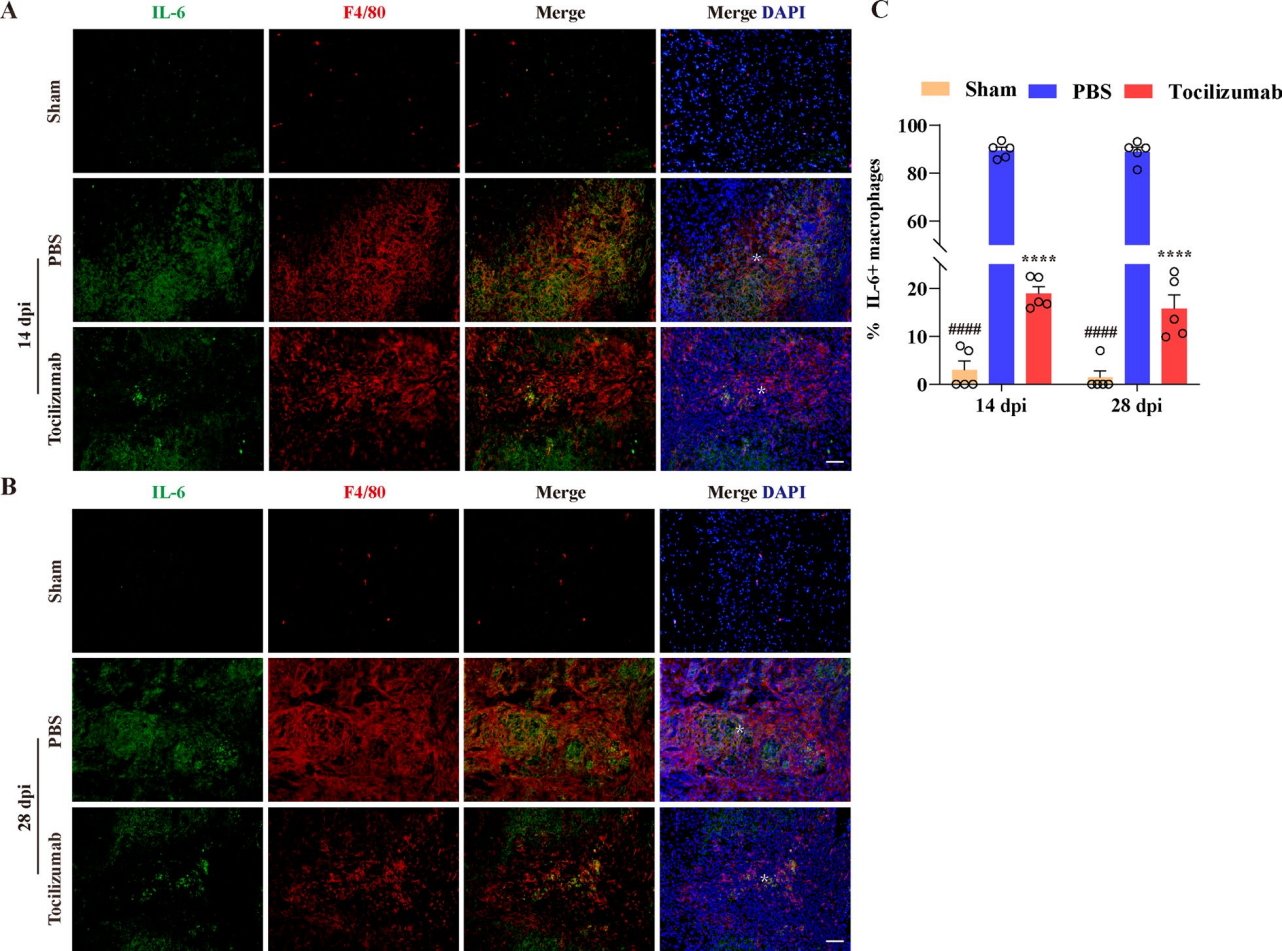
Tocilizumab reduces expression of IL-6 in macrophages in the lesion epicenter after SCI. **A**, **B** Representative images of the spinal cords of the sham, PBS, and Tocilizumab groups are stained for IL-6 (green) and F4/80 (red) at 14 (**A**) and 28 dpi (**B**), which exhibits most colocalization of F4/80 and IL-6 in the injured core in the PBS group, while less colocalization of F4/80 and IL-6 in the Tocilizumab group. Asterisks indicate the injured core. Scale bar: 100 μm. **B** Quantification of the IL6^+^ macrophages at 14 and 28 dpi. Data are presented as the mean ± SEM (n = 5 in each group). ****p < 0.0001 (Tocilizumab vs. PBS). ^####^*p* < 0.0001 (sham vs. PBS and Tocilizumab)

Tocilizumab has been reported to reduce the expression of IL-6 by blocking IL-6R [26, 27]. To further confirm the effect of Tocilizumab treatment on the expression of IL-6 in macrophages in the lesion epicenter after SCI, we performed immunofluorescence staining of F4/80 and IL-6 in injured spinal cord tissues at 14 and 28 dpi. As shown in Fig. 6A, B, the proportion of IL-6^+^ or F4/80^+^ macrophages were rare low in the sham group spinal cord. By contrast, the colocalization of F4/80 and IL-6 in the injured core was increased significantly at 14 and 28 dpi as presented in the PBS group. Tocilizumab treatment hindered the ability of macrophages to express IL6 (Fig. 6A–C). These results reveal that Tocilizumab treatment can decrease the expression of IL-6 in macrophages in the lesion epicenter after SCI.

## Discussion

The expression of IL-6 in spinal cord tissue was significantly increased and played a proinflammatory role after SCI [28]. Inhibition of IL-6 is beneficial for reducing extensive inflammation after SCI in mice [9]. However, research on the effect of inhibiting IL-6 on TJs restoration in the BSCB has not been reported. Our present study confirms that Tocilizumab which blocks IL-6R can promote the restoration of TJs between vascular endothelial cells, reduce BSCB leakage and the expression of IL-6 in macrophages, and facilitate axon regeneration and motor functional recovery after SCI.

Depleting macrophages after SCI is beneficial to the repair of the BSCB, but we found that depleting macrophages had no obvious effect on axon regeneration and functional recovery after SCI. This may be related to the elimination of the beneficial effects of macrophages on axon regeneration. The role of CL in depleting macrophages is extensive and CL cannot specifically deplete macrophages in a certain polarization. The beneficial effects of M2 macrophages on axon regeneration and tissue repair have been confirmed [29]. The depletion of M2 macrophages by CL may be one of the reasons for the inhibition of axon regeneration. The depletion of macrophages is also unfavorable for the clearance of myelin debris in the injured site. The persistent presence of myelin debris aggravates the inflammatory response and hinders the regeneration of axons [30]. Moreover, macrophages can also facilitate angiogenesis. Depletion of macrophages after skin injury reduces the stability of new blood vessels, which is not conducive to angiogenesis [31]. Zhu et al. applied CL to deplete macrophages after SCI and found that the number of lectin-labelled blood vessels in the injured core was significantly decreased at 14 dpi after SCI, indicating that macrophages in the injured core were beneficial to endogenous angiogenesis [32]. Therefore, the depletion of macrophages may lead to insufficient blood supply, resulting in impaired axonal regeneration. Lee’s study demonstrated that the continuous depletion of macrophages had no obvious effect on axon regeneration at 14 dpi but promoted axon regeneration in the injured core at 56 dpi after SCI [32]. M2 macrophages exist for a short time after SCI, while M1 macrophages can persist in the injured site [33]. Hence, sustained clearance of macrophages may mainly deplete M1 macrophages, thereby alleviating the detrimental effects of M1 macrophages, which may be responsible for promoting axon regeneration in the chronic phase of SCI.

Within 24 h after SCI, the increased expression of IL-6 in the spinal cord parenchyma can be detected [34]. IL-6 binds to its receptor to form an IL-6/IL-6R complex, which further binds to intracellular glycoprotein 130 (gp130) and activates downstream pathways [35]. IL-6 plays a role in recruiting neutrophils, lymphocytes, monocytes, and other immune cells and activates astrocytes and microglia to promote inflammatory responses and glial scar formation after SCI [36]. Low concentrations of IL-6 can induce the expression of neurotrophic factors and promote the activation and recruitment of leukocytes, which is beneficial to the clearance of myelin debris and axon regeneration. However, high concentrations of IL-6 induce inflammatory cells to secrete cyclooxygenase-2 (COX-2), inducible nitric oxide synthase (iNOS) and other neurotoxic substances, which exacerbate tissue injury [28]. Blocking IL-6R with MR16-1 can reduce the inflammatory response and ameliorate functional recovery after SCI in mice [8, 9].

An in vitro study demonstrated that IL-6 can cause redistribution of endothelial ZO-1 and actin via the protein kinase C pathway and reduce transendothelial resistance between endothelial cells, resulting in barrier dysfunction of endothelial cells [37]. In addition, by applying an anti-IL-6R neutralizing antibody in vitro, Blecharz-Lang et al. found that antagonizing IL-6R can effectively increase the expression of ZO-1 and occludin in endothelial cells [38]. Our previous study suggested that macrophages aggregated in the injured cord after SCI impaired endothelial TJs by secreting IL-6, leading to persistent leakage of the BSCB [7]. In the present study, we specifically blocked IL-6R in mice by intrathecal injection of Tocilizumab and found that the expression of IL-6 in macrophages was significantly decreased and the expression of endothelial ZO-1 and CLDN5 was significantly increased at 14 and 28 dpi, accompanied by decreased leakage of the BSCB, suggesting that Tocilizumab can effectively promote the restoration of the BSCB after SCI. These results suggests that Tocilizumab may promote the restoration of TJs between vascular endothelial cells after SCI by reducing the IL-6 secretion of macrophages in the injured core. CD11b^+^ cells in the injured core were significantly decreased at 14 dpi by blocking the IL-6R in mice, which demonstrated that antagonizing IL-6R can reduce the inflammatory response in the injured core after SCI [9]. In this study, we found that CD68^+^ macrophages/microglia aggregated in the injured core were significantly decreased at 14 and 28 dpi after continuous intrathecal injection of Tocilizumab, confirming that Tocilizumab can reduce the infiltration of macrophages/microglia in the injured core after SCI. This finding suggests that in addition to directly inhibiting the destructive effect of IL-6 on TJs between vascular endothelial cells, Tocilizumab may also promote the restoration of TJs by reducing the infiltration of macrophages in the injured core after SCI. Furthermore, the polarization of macrophages may be affected by the antagonism of IL-6R. Guerrero et al. administered a single intraperitoneal injection of MR16-1 to SCI mice and found that the expression of iNOS and CD16/32 in macrophages decreased at 3, 7, and 14 dpi after SCI, while the expression of arginase 1 (Arg-1) and CD206 increased, which indicated that the polarization of macrophages could be converted from M1 to M2 after antagonizing IL-6R [39]. M1 macrophages can impair endothelial TJs whereas M2 macrophages protect endothelial TJs in an inflammatory state [7]. Therefore, Tocilizumab may also promote the restoration of TJs between vascular endothelial cells after SCI by regulating the polarization state of macrophages, which remains to be further explored.

Depletion of macrophages did not significantly facilitate axon regeneration and motor function recovery after SCI, while intrathecal injection of Tocilizumab significantly promoted axon regeneration and motor functional recovery in the chronic phase of SCI. This is inseparable from the specific blocking effect of Tocilizumab on IL-6R, which promotes the repair of the BSCB and is beneficial for reducing inflammation. Blockade of IL-6R by MR16-1 attenuates the inflammatory response in the injured site and promotes functional recovery at 42 dpi after SCI [8]. Further study found that a single intraperitoneal injection of MR16-1 attenuated macrophage-induced inflammatory responses and increased microglia-dominated inflammation, resulting in axon regeneration at 42 dpi after SCI [9]. Through continuous intrathecal injection of Tocilizumab, we found that it exhibited a significant effect on promoting axon regeneration at 14 dpi. In addition, the improvement of functional recovery had already occurred at 21 dpi after SCI. The effective time was earlier than that of a single injection of MR16-1 after SCI to facilitate axon regeneration and functional recovery, which may be due to the sustained inhibition of the proinflammatory effect of IL-6 by continuous intrathecal administration. Tocilizumab also reduces neuronal death in subarachnoid haemorrhage rats [40]. The antagonism of IL-6R after SCI improves the survival of transplanted bone marrow stem cells [41]. These studies indicate that in the central nervous system, Tocilizumab may have a protective effect on cell survival. This may be one of the mechanisms by which Tocilizumab is beneficial to axon regeneration after SCI. In this study, we also found that Tocilizumab reduced the formation of fibrotic scar in the chronic phase of SCI, which is likely also related to the reduction in macrophage infiltration [32]. The reduction in fibrotic scarring may also be one of the reasons for the promotion of axon regeneration.

## Conclusion

In conclusion, our study demonstrates that Tocilizumab can reduce the IL-6 expression of macrophages in injured core and promote the recovery of TJs between vascular endothelial cells and axon regeneration in mice after SCI. Tocilizumab is a potential effective drug for the treatment of reconstructive BSCB function after SCI.

## Supplementary Information

**Additional file 1: Figure S1.** Depletion of macrophages has no effect on axonregeneration and motor functional recovery after SCI. **A** A schematicillustration of the CL injection protocol for depleting macrophages after SCI.PL or CL are stereotaxically injected into the lesion site at 5 dpi and the effectof macrophage depletion is assessed at 14 dpi after SCI. **B** Representativeimages of sham, PL, and CL groups are stained for CD68 at 14 dpi, which exhibitfew CD68^+^ cells in the injured corein the CL group. **C** Representative images of sham, PL, and CL groupsare stained for NF (red) and GFAP (green) at 14 dpi, whichexhibit few NF^+^ axons in the lesion epicenter in the PL and CL groups.Asterisks indicate the injuredcore. **D** Quantitative analysis of the number of NF^+^axons in the GFAP^−^ region at 14 dpi. Data are presented as the mean ± SEM (n = 5 in each group). **E** The time course of functional recoverybased on the BMS scores in the sham, PL, and CL groups. Data are presentedas mean ± SEM (n = 8 in each group). **F**–**I** The results and quantitationof footprint analyses in the sham, PL, and CL groups at 28 dpi after SCI. Data are presentedas the mean ± SEM (n = 8 in each group). ^###^*p* < 0.001 and ^####^*p* < 0.0001 (sham vs. PL and CL). ns, no significance(PL vs. CL). Scale bars: 100 μm (**B**, **C**).

## Data Availability

The data used and/or analyzed during the current study are available from the corresponding author on reasonable request.

## Abbreviations

TJs: Tight junctions
IL-6: Interleukin-6
SCI: Spinal cord injury
IL-6R: IL-6 receptor
BSCB: Blood-spinal cord barrier
dpi: Days postinjury
CLDN5: Claudin-5
ZO-1: Zonula occludens-1
FDA: Food and Drug Administration
PBS: Phosphate buffered saline
GFAP: Glial fibrillary acidic protein
NF: Neurofilament
GAP-43: Growth-associated protein 43
HE: Hematoxylin and Eosin
CL: Clodronate liposomes
PL: PBS liposomes
SEM: Standard error of the mean
COX-2: Cyclooxygenase-2
iNOS: Inducible nitric oxide synthase
Arg-1: Arginase 1
