# Supplementary material for: Tocilizumab promotes repair of spinal cord injury by facilitating the restoration of tight junctions between vascular endothelial cells
Source: Fluids Barriers CNS. 2023 Jan 9;20:1. doi: 10.1186/s12987-022-00399-9 (PMC9830903; doi:10.1186/s12987-022-00399-9)
Supplement: Supplementary file 1 — Additional file 1: Figure S1. Depletion of macrophages has no effect on axonregeneration and motor functional recovery after SCI. A A schematicillustration of the CL injection protocol for depleting macrophages after SCI.PL or CL are stereotaxically injected into the lesion site at 5 dpi and the effectof macrophage depletion is assessed at 14 dpi after SCI. B Representativeimages of sham, PL, and CL groups are stained for CD68 at 14 dpi, which exhibitfew CD68+ cells in the injured corein the CL group. C Representative images of sham, PL, and CL groupsare stained for NF (red) and GFAP (green) at 14 dpi, whichexhibit few NF+ axons in the lesion epicenter in the PL and CL groups.Asterisks indicate the injuredcore. D Quantitative analysis of the number of NF+axons in the GFAP− region at 14 dpi. Data are presented as the mean ± SEM (n = 5 in each group). E The time course of functional recoverybased on the BMS scores in the sham, PL, and CL groups. Data are presentedas mean ± SEM (n = 8 in each group). F–I The results and quantitationof footprint analyses in the sham, PL, and CL groups at 28 dpi after SCI. Data are presentedas the mean ± SEM (n = 8 in each group). ###p < 0.001 and ####p < 0.0001 (sham vs. PL and CL). ns, no significance(PL vs. CL). Scale bars: 100 μm (B, C). [file 12987_2022_399_MOESM1_ESM.docx]

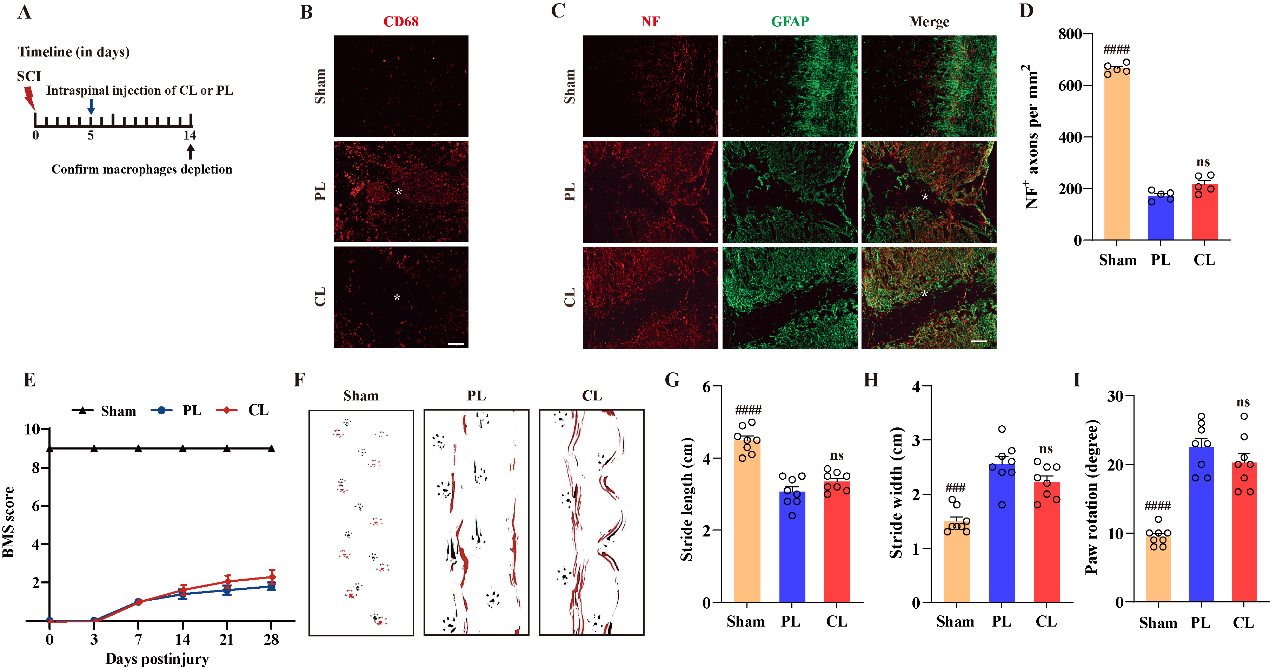


**Fig. S1.** Depletion of macrophages has no effect on axon regeneration and motor functional recovery after SCI. **(A)** A schematic illustration of the CL injection protocol for depleting macrophages after SCI. PL or CL are stereotaxically injected into the lesion site at 5 dpi and the effect of macrophage depletion is assessed at 14 dpi after SCI. **(B)** Representative images of sham, PL, and CL groups are stained for CD68 at 14 dpi, which exhibit few CD68^+^ cells in the injured core in the CL group. **(C)** Representative images of sham, PL, and CL groups are stained for NF (red) and GFAP (green) at 14 dpi, which exhibit few NF^+^ axons in the lesion epicenter in the PL and CL groups. Asterisks indicate the injured core. **(D)** Quantitative analysis of the number of NF^+^ axons in the GFAP^−^ region at 14 dpi. Data are presented as the mean ± SEM (n = 5 in each group). **(E)** The time course of functional recovery based on the BMS scores in the sham, PL, and CL groups. Data are presented as mean ± SEM (n = 8 in each group). **(F-I)** The results and quantitation of footprint analyses in the sham, PL, and CL groups at 28 dpi after SCI. Data are presented as the mean ± SEM (n = 8 in each group). ^###^*p* < 0.001 and ^####^*p* < 0.0001 (sham vs. PL and CL). ns, no significance (PL vs. CL). Scale bars: 100 μm (**B** and **C**).
